# Treatment of Ostial Right Coronary Artery Narrowings: Outcomes From the Multicenter Prospective e-ULTIMASTER Registry

**DOI:** 10.1016/j.jscai.2023.100604

**Published:** 2023-02-25

**Authors:** Yaniv Levi, Ofer Kobo, Majdi Halabi, Imad Al Haddad, Bernard Chevalier, Jawed Polad, Peep Laanmets, Adam Witkowski, Jacques Monsegu, Andres Romo Iniguez, Mamas A. Mamas, Ariel Roguin, e-ULTIMASTER Registry Investigators

**Affiliations:** a Department of Cardiology, Hillel Yaffe Medical Center, Technion-Faculty of Medicine, Hadera, Israel; b Department of Cardiology, Ziv Hospital, Safed, Israel; c Cardiovascular Division, Department of Medicine, Jordan Hospital, Amman, Jordan; d Institut Cardiovasculaire Paris Sud, Hôpital Privé Jacques Cartier, Massy, France; e Department of Cardiology, Jeroen Bosch Ziekenhuis, Hertogenbosch, the Netherlands; f Department of Cardiology, North Estonia Medical Centre, Tallinn, Estonia; g Department of Interventional Cardiology and Angiology, Institute of Cardiology, Warsaw, Poland; h Institut Cardio-Vasculaire, Groupe Hospitalier Mutualiste, Grenoble, France; i Department of Cardiology, Hospital Álvaro Cunqueiro, Vigo, Spain; j Keele Cardiovascular Research Group, Centre for Prognosis Research, Institutes of Applied Clinical Science and Primary Care and Health Sciences, Keele University, Keele, Newcastle, United Kingdom

**Keywords:** ostial lesion, percutaneous coronary intervention, right coronary artery

## Abstract

**Background:**

Treatment of right coronary artery (RCA) aorto-ostial (AO) lesions with bare-metal stents and first-generation drug-eluting stents (DES) was associated with worse outcomes. This study aimed to assess the effect of RCA-AO stenting with current-generation DES on the clinical outcome.

**Methods:**

The large all-comer, multicontinental e-ULTIMASTER registry included 37,198 patients of whom 4775 underwent ostial and proximal RCA percutaneous coronary intervention (PCI) using the Ultimaster stent (Terumo). The primary clinical end point was 1-year target lesion failure (TLF), a composite of cardiac death; target vessel–related myocardial infarction; or clinically indicated target lesion revascularization.

**Results:**

We compared 591 (12.4%) patients who underwent RCA-AO PCI with 4184 (87.6%) patients who underwent proximal RCA PCI. The RCA-AO group included more men and recorded significantly more comorbidities and more complex coronary anatomy. After propensity matching, the primary end point TLF occurred in 4.49% of the RCA-AO group compared with 3.00% of the proximal RCA group (*P* = .06). Target vessel revascularization (3.29% vs 1.90%; *P* = .03) and stent thrombosis (1.23% vs 0.42%, *P* = .01) were significantly higher among patients with RCA-AO lesions than those among patients with proximal RCA lesions. All-cause mortality was similar between the groups (2.97% vs 2.26%; *P* = .30).

**Conclusions:**

The treatment of RCA-AO with DES is feasible, with similar rates of TLF but with an increased risk of target vessel revascularization and stent thrombosis.

## Highlights

• The largest study to date, accessing the clinical outcome of RCA aorto-ostial stenting with current DES.
• Compared with the proximal RCA, the treatment of RCA aorto-ostial is feasible, with similar rates of target lesion failure.
• RCA aorto-ostial PCI was associated with an increased risk of target vessel revascularization and stent thrombosis.

## Central Illustration

**Figure:**
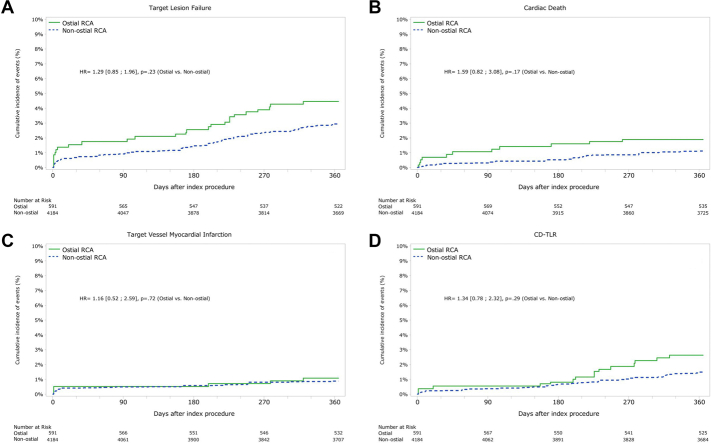

## Introduction

The treatment of ostial coronary narrowings is challenging. Percutaneous coronary intervention (PCI) of right coronary artery (RCA) aorto-ostial (AO) lesions, using balloon angioplasty, bare-metal stents, or first-generation drug-eluting stents (DES), showed higher restenosis rates and target lesion failure (TLF) than that of other RCA lesions.^1^, ^2^, ^3^, ^4^ PCI with DES compared with that using bare-metal stents was associated with improved outcomes in the treatment of AO lesions. Small studies have suggested that new-generation drug-eluting stents (nDES) have better outcomes than early-generation drug-eluting stents (eDES) in the treatment of RCA-AO lesions.^4^, ^5^, ^6^, ^7^ Currently, data regarding the outcome of RCA-AO PCI with DES are limited. Therefore, we aimed to compare the immediate results and follow-up outcomes of RCA-AO lesions with those of proximal RCA lesions treated with contemporary generation DES.

## Methods

The e-ULTIMASTER registry is an all-comer patient population with an indication for PCI, which enrolled 37,198 patients. This study analyzed the clinical outcomes of patients enrolled in the e-Ultimaster registry divided according to whether stents were implanted in RCA-AO versus proximal RCA segments. RCA-AO lesions were defined within 3.0 mm of the aorto-ostium. There were 10 patients with both RCA-AO and proximal RCA lesions, and they were excluded from the analysis.

The inclusion and exclusion criteria^8^^,^^9^ were minimal to better evaluate the Ultimaster stent (Terumo). Sites in Europe, Asia, Africa, Middle East, South America, and Mexico participated in the registry, using a thin-strut (80.0 μm) cobalt-chromium sirolimus-eluting stent (SES). This stent features a biodegradable polymer coating (poly-d,l-lactic acid polycaprolactone) that is fully metabolized through *dl*-lactide and caprolactone into carbon dioxide and water in 3 to 4 months. This coating is applied on the abluminal side of the struts only; after resorption, a bare-metal stent remains.

The primary end point was 1-year TLF, defined as the composite of cardiac death, target vessel–related myocardial infarction (TVMI), and clinically driven target lesion revascularization (TLR). The patient-oriented composite end point was defined as all-cause death, any myocardial infarction (MI), and any revascularization (Supplemental Table 1). Major adverse cardiac events were defined as cardiac death, any MI, and any revascularization. For MI, the extended historical MI definition was applied, which primarily uses the creatine kinase myocardial band, or, if not available, troponin, as a cardiac biomarker criterion. Major bleeding was defined as a Bleeding Academic Research Consortium (BARC) 3 or 5 bleeding. Stent thrombosis was defined according to the Academic Research Consortium definitions at the 3-month and 1-year follow-up.

All primary end point–related events were adjudicated by an independent clinical events committee (Supplemental Table 2). The data that support the findings of this study are available from the corresponding author upon reasonable request. The study was approved by the ethical committees of the participating sites, and all patients provided written informed consent (Supplemental Table 3). The ClinicalTrials.gov identifier was NCT02188355.

Follow-up was performed at 3 months and 1 year after the index procedure. Patients were contacted by telephone or by a visit to the outpatient clinic. Relevant information on adverse events, such as death, MI, re-PCI, coronary artery bypass grafting (CABG), bleeding, vascular complications, or stent thrombosis, were collected.

### Statistical analysis

Baseline characteristics were reported as mean ± SD for continuous variables and number and percentages for categorical variables. Statistical differences between baseline characteristics were reported using the *t* test for continuous variables and χ^2^ test for categorical variables.

To reduce the effect of baseline differences between the 2 groups, a propensity-score analysis was performed using separate models for the lesion location. Propensity scores were calculated using a logistic regression model, with the subgroup (RCA-AO or proximal RCA) as the outcome and the variables that needed to be matched for as independent variables. The probability of belonging to 1 of the 2 groups was used as a propensity score. Variables to be entered into the model were predefined based on any possible effect on the outcomes and included the following: age, sex, diabetes mellitus, hypertension, renal impairment, a history of MI, previous PCI, previous CABG, previous stroke, peripheral vascular disease, ST-segment elevation myocardial infarction (STEMI), non-STEMI, acute coronary syndrome, radial access, index target vessel left main coronary artery, number of lesions treated, total length of successfully implanted stents, and type B2 and C lesions according to the American College of Cardiology/American Heart Association classification. The inverse probability of the treatment weight methodology was used to perform a matched analysis. This methodology uses the inverse of the propensity score of its own subgroup (ie, the probability of the subject of belonging to the subgroup) as a weight that can be used in the analyses. The balance after matching can be tested by calculating the weighted standardized difference for the inverse probability of treatment weight analysis using the calculated weights. Generally, standardized differences for all variables <0.20 are considered well balanced, whereas standardized difference for all variables <0.10 can be considered extremely well balanced. Weighted χ^2^ tests were used for binary or categorical data; weighted Wilcoxon rank-sum tests were used for continuous data. For the primary outcome TLF, a stepwise selected model was used, where all variables are entered 1 by 1 if their *P* value is <0.25 (entry *P* value) and retains the variables in the next steps when the *P* value remains <0.15 (stay *P* value). This gave a model with only a set of important, predictive variables and avoided multicollinearity. *P* <.05 was considered significant. Statistical analyses were performed using SAS software version 9.4 (SAS Institute).

## Results

### Study patients

**Figure 1:**
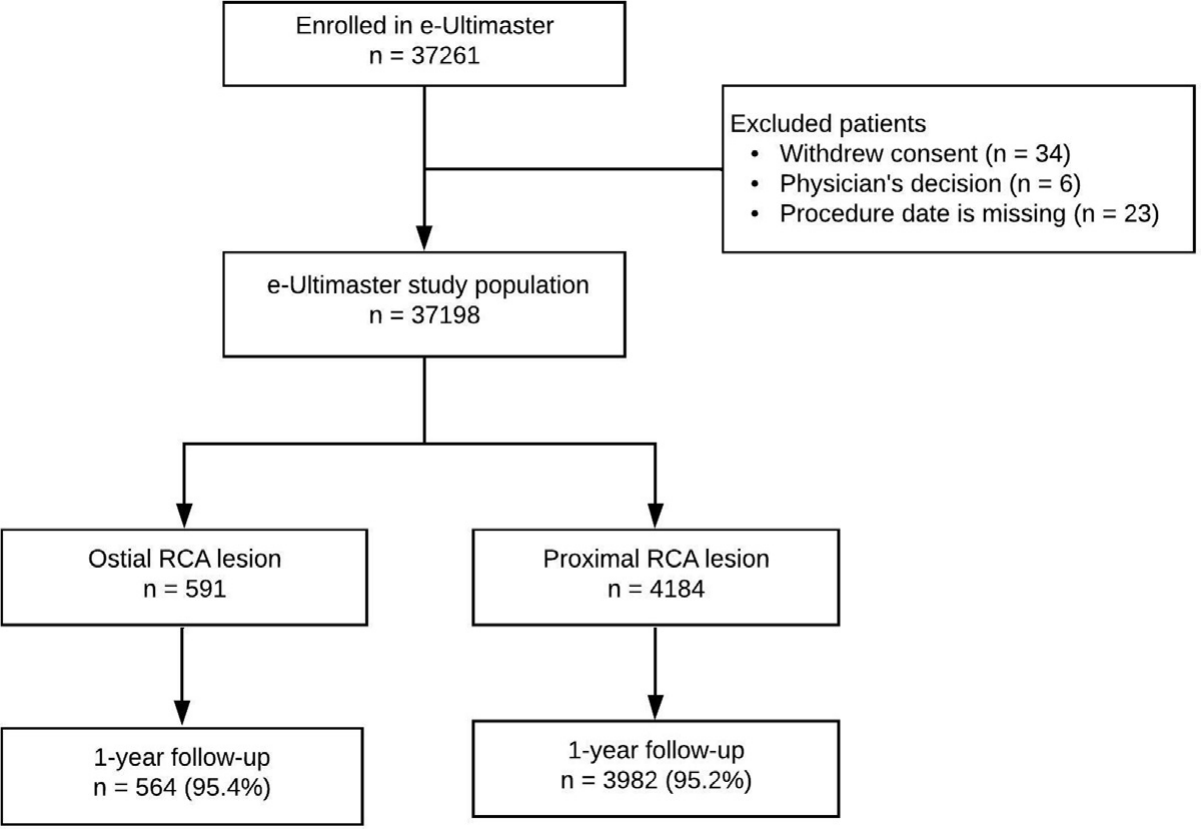
**Study patients.** RCA, right coronary artery.

A total of 37,198 patients who underwent PCI in 413 sites over 50 countries were included in our analysis. Enrollment ended in May 2018. This analysis included 4775 patients. We compared patients with a stent in the RCA-AO segment (n = 591) with those with a stent in the proximal segment of the RCA (n = 4184). Overall, 4546 patients completed 1-year follow-up data: 564 (95.4%) in the RCA-AO group and 3982 (95.2%) in the proximal RCA group (Figure 1).

### Baseline demographics

The baseline characteristics comparing the 2 groups are presented in Table 1. Patients in the RCA-AO group were older, with a higher proportion of women, higher prevalence of hypertension, peripheral vascular disease, renal impairment, and a history of stroke, MI, PCI, or CABG. They presented with a higher incidence of silent ischemia and stable angina, whereas patients in the proximal RCA group recorded a higher incidence of acute coronary syndrome, unstable angina, and STEMI. Table 2 presents the baseline lesions and procedural characteristics. The number of treated lesions, number of stents implanted and total stent length was higher among patients in the RCA-AO group.

**Table 1:** Baseline patient characteristics.

	RCA non-ostial (n = 4184)	RCA-AO (n = 591)	*P*
Age, y	64.4 ± 11.21	68.7 ± 10.45	<.0001
Male sex	75.05 (3140/4184)	62.10 (367/591)	<.0001
Diabetes mellitus	28.22 (1158/4104)	30.09 (176/585)	.35
Hypertension	68.61 (2579/3759)	75.04 (418/557)	<.01
Hypercholesterolemia	61.73 (2249/3643)	65.19 (354/543)	.12
Family history of heart disease	37.42 (824/2202)	35.31 (119/337)	.46
Previous stroke	4.49 (174/3875)	7.72 (44/570)	<.001
Peripheral vascular disease	7.45 (283/3797)	14.18 (80/564)	<.0001
LVEF, %	53.7 ± 11.48 (1695)	54.1 ± 10.46 (264)	.59
Renal impairment	6.96 (284/4080)	12.54 (73/582)	<.0001
Previous myocardial infarction	25.23 (978/3876)	29.38 (166/565)	.04
Previous PCI	26.98 (1054/3907)	39.97 (229/573)	<.0001
Previous CABG	4.70 (183/3893)	8.04 (46/572)	.001
Silent ischemia	8.64 (361/4180)	11.17 (66/591)	.04
Stable angina	35.02 (1464/4180)	40.10 (237/591)	.02
Acute coronary syndrome	56.34 (2355/4180)	48.73 (288/591)	<.001
Unstable angina	11.32 (473/4180)	14.89 (88/591)	.01
NSTEMI	20.72 (866/4180)	20.30 (120/591)	.82
STEMI	24.28 (1015/4180)	13.54 (80/591)	<.0001

Values are mean ± SD or % (n/N).

AO, aorto-ostial; CABG, coronary artery bypass graft; LVEF, left ventricular ejection fraction; NSTEMI, non–ST-segment elevation myocardial infarction; PCI, percutaneous coronary intervention; RCA, right coronary artery; STEMI, ST-segment elevation myocardial infarction.

**Table 2:** Baseline lesion and procedural characteristics.

	RCA non-ostial (n = 4184)	RCA-AO (n = 591)	*P*
No. of lesions treated at index procedure	1.6 ± 0.81 (4181)	1.7 ± 0.90 (591)	<.001
RCA treated	100.0 (4184/4184)	100.0 (591/591)	
LAD artery treated	16.20 (678/4184)	15.74 (93/591)	.77
LCX artery treated	11.28 (472/4184)	11.17 (66/591)	.93
Arterial or venous bypass graft treated	0.17 (7/4184)	0.17 (1/591)	.99
Left main treated	1.08 (45/4184)	3.72 (22/591)	<.0001
Femoral access	22.75 (952/4184)	28.43 (168/591)	<.01
Radial access	79.76 (3337/4184)	75.80 (448/591)	.03
Brachial access	0.38 (16/4184)	0.17 (1/591)	.42
Ostial lesion (<3.0 mm) RCA	0.00 (0/4207)	100.00 (591/591)	<.0001
No. of lesions treated per patient	1.6 ± 0.81 (4181)	1.7 ± 0.90 (591)	<.001
No. of stents successfully implanted per patient	2.0 ± 1.12 (4178)	2.1 ± 1.23 (591)	<.001
Total length of stents successfully implanted/patient, mm	40.9 ± 26.59 (4170)	38.3 ± 29.11 (588)	.03
Stent diameter/patient, mm	3.3 ± 0.46 (4170)	3.4 ± 0.48 (588)	<.0001
Procedural details for proximal or ostial RCA			
Atherectomy	0.90 (38/4207)^a^	2.37 (14/591)	<.01
Cutting balloon	1.00 (42/4207)^a^	4.23 (25/591)	<.0001
Predilation	59.78 (2515/4207)^a^	65.65 (388/591)	<.01
Postdilation	44.50 (1872/4207)^a^	52.12 (308/591)	<.01
No. of stents successfully implanted	1.3 ± 0.66 (4165)	1.3 ± 0.65 (583)	.27
Total length of stents successfully implanted, mm	31.5 ± 19.64 (3847)	26.4 ± 19.06 (533)	<.001
Stent diameter, mm	3.4 ± 0.40 (3847)	3.5 ± 0.42 (533)	<.001

Values are mean ± SD or % (n/N).

AO, aorto-ostial; LAD, left anterior descending; LCX, left circumflex; RCA, right coronary artery.

a The number of patients with 2 lesions in the proximal segment of the RCA.

### Clinical outcomes

**Figure 2:**
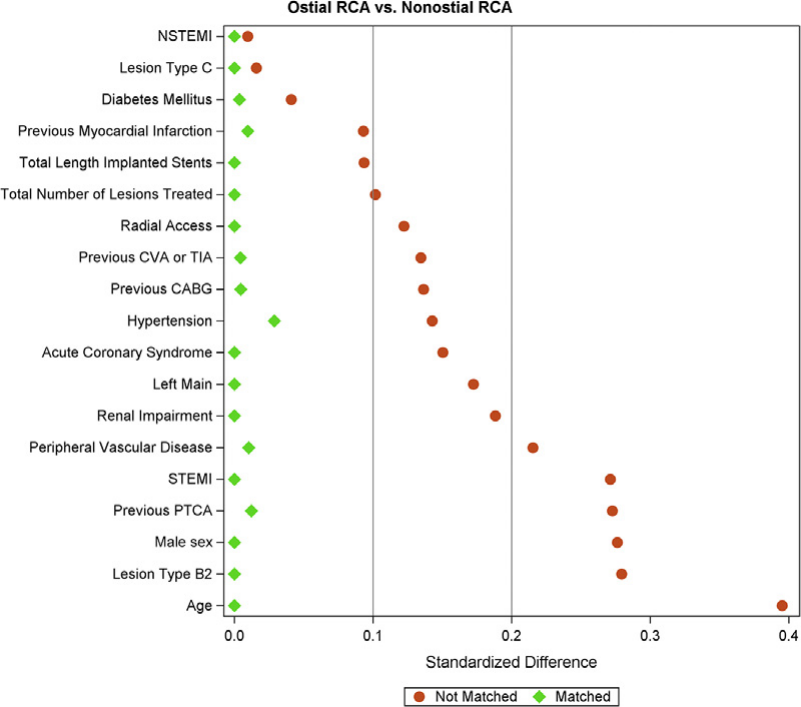
**Standardized differences in variables included in the propensity score between the groups**. After adjustment, all covariates in the planned propensity score had weighted standardized differences of <0.1, which indicates an equilibration of these covariates between the groups. CA, cardiovascular arteries; CABG, coronary artery bypass grafting; CAS, acute coronary syndrome; CVA, cerebrovascular attack; LM, left main coronary artery; NSTEMI, non–ST-segment elevation myocardial infarction; PTCA, percutaneous transluminal coronary angioplasty; RCA, right coronary artery; RIND, reversible ischemic neurological deficit; STEMI, ST-segment elevation myocardial infarction; TIA, transient ischemic attack.

Nonadjusted, and adjusted 1-year follow-up event rates are presented in Table 3. After inverse propensity-score weighing (IPSW), the primary end point of TLF was numerically higher with RCA-AO lesions and did not reach a significant difference (4.49% vs 3.00%; *P* = .06, for RCA-AO vs proximal RCA lesions). We found no significant difference in the primary outcome components of cardiac death (1.90% vs 1.15%; *P* = .13), TVMI (1.09% vs 0.91%; *P* = .69), and clinically driven TLR (2.61% vs 1.5%; *P* = .05) for the RCA-AO group and proximal RCA groups. The Central Illustration shows the cumulative event incidence by the Kaplan-Meier method of TLF and its components after IPSW (Figure 2).

**Table 3:** Unadjusted and propensity-weighted clinical outcomes.

	Unadjusted			Propensity-weighted		
	RCA non-ostial (n = 3982)	RCA-AO (n = 564)	*P*	RCA non-ostial (n = 3982)	RCA-AO (n = 564)	*P*
Target lesion failure	2.41 (96/3982)	4.61 (26/564)	<.01	3.00 (119/3982)	4.49 (25/564)	.06
Target vessel failure	2.79 (111/3982)	5.14 (29/564)	<.01	3.37 (134/3982)	5.04 (28/564)	.05
POCE	5.95 (237/3982)	8.69 (49/564)	.01	6.84 (272/3982)	8.52 (48/564)	.15
All death	1.71 (68/3982)	3.19 (18/564)	.02	2.26 (90/3982)	2.97 (17/564)	.30
Cardiac death	0.85 (34/3982)	1.95 (11/564)	.01	1.15 (46/3982)	1.90 (11/564)	.13
All myocardial infarctions	0.93 (37/3982)	1.77 (10/564)	.06	1.19 (47/3982)	1.82 (10/564)	.22
Target vessel–related myocardial infarction	0.75 (30/3982)	1.06 (6/564)	.44	0.91 (36/3982)	1.09 (6/564)	.69
Clinically driven target vessel revascularization	1.71 (68/3982)	3.37 (19/564)	.01	1.90 (76/3982)	3.29 (19/564)	.03
Clinically driven target lesion revascularization	1.28 (51/3982)	2.66 (15/564)	.01	1.50 (60/3982)	2.61 (15/564)	.05
Clinically driven target vessel nontarget lesion revascularization	0.50 (20/3982)	0.71 (4/564)	.53	0.54(21/3982)	0.68(4/564)	.67
Stent thrombosis, definite/probable	0.55 (22/3982)	1.24 (7/564)	.05	0.42(17/3982)	1.23(7/564)	.01
Bleeding or vascular complication	3.64 (145/3982)	5.67 (32/564)	.02	3.79 (151/3982)	4.77 (27/564)	.26
BARC 3-5 bleeding	1.08 (43/3982)	1.42 (8/564)	.47	1.39 (55/3982)	1.27 (7/564)	.82

AO, aorto-ostial; BARC, Bleeding Academic Research Consortium; POCE, patient-oriented composite end point; RCA, right coronary artery.

**Central Illustration:**
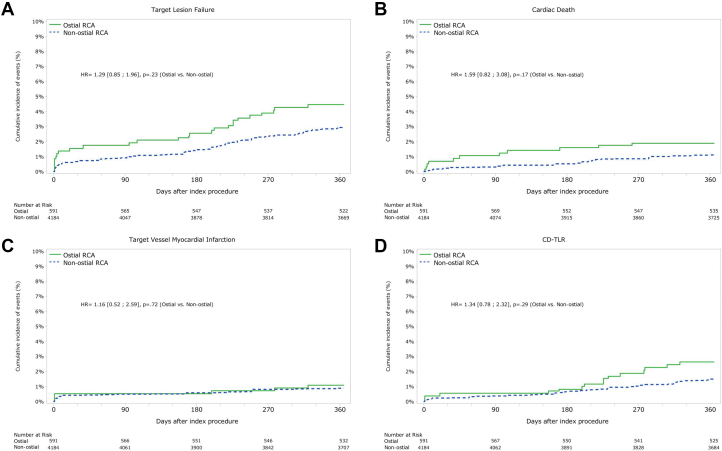
**Cumulative event incidence by Kaplan-Meier method after inverse propensity-score weighing**. (A) Target lesion failure, (B) cardiac death, (C) target vessel–related myocardial infarction, and (D) clinically driven target lesion revascularization. CD-TLR, clinically driven target lesion revascularization; RCA, right coronary artery.

In the RCA-AO group, we found a higher incidence of clinically driven TVR (3.29% vs 1.9%; *P* = .03) and stent thrombosis rates (1.23% vs 0.42%, *P* = .01). At hospital discharge, 97.7% and 96.8% (*P* = .20) received dual antiplatelet therapy in the proximal RCA and RCA-AO groups, respectively.

## Discussion

In this large study using DES that are currently in clinical use, we found that the primary outcome of TLF was similar when the narrowing involved the ostium of RCA lesions compared with that of proximal RCA lesions. There was no difference in mortality or in MI. The size of the subgroup of patients with PCI-treated RCA-AO lesions is the largest reported so far. Of note, there was a significantly higher clinically driven TVR and stent thrombosis in RCA-AO–treated lesions than in proximal RCA-treated lesions. Table 4 compares the outcomes of previously reported RCA-AO PCI studies.

**Table 4:** Comparisons between previously reported right coronary artery-aorto-ostial percutaneous coronary intervention outcomes.

Study	AO (n)	NO (n)	SG	AO Def	Follow-up (y)	TLR (%) AO	TLR (%) NO	ST (%) AO	ST (%) NO	All-cause death AO (%)	All-cause death NO (%)
Sakamoto et al^10^	37 DES 36 BMS	62 DES	First	3 mm	0.7	13.5 36.1	1.61	0 0	0	0 0	0
Al-Lamee et al^5^	230 DES 116 BMS		First and second	3 mm	3.3 2.5	12 27		4 1		11 18	
Ko et al^3^	397	3716	First	3 mm	5	28.2	13.7	2	1.3	25.7	14.4
Lam et al^4^	67	254	Second	3 mm	2	7.5	1.6	1.5	1.6	7.5	2.4
Watanabe et al^6^	76 eDES 74 nDES			3 mm	2	43.1 12.7		0 0		2.7 5.4	
Mitomo et al^7^	142 eDES 192 nDES			3 mm	3	38 11		0 0		- -	
Current study	591	4184	Third	3 mm	1	4.49	3	1.23	0.42	2.97	2.26

AO, aorto-ostial; BMS, bare-metal stent; Def, definition; DES, drug-eluting stent; eDES, early drug-eluting stent; nDES, new drug-eluting stent; NO, non-ostial; PCI, percutaneous coronary intervention; RCA, right coronary artery; SG, stent generation; ST, stent thrombosis; TLR, target lesion revascularization.

Data regarding the RCA-AO revascularization, especially in the nDES are lacking. Only few studies compared the treatment of RCA-AO lesions with that of non-ostial RCA lesions. Sakamoto et al^10^ found a marked increase in TLR in RCA-AO lesions treated by first-generation SES, compared with that in non-ostial RCA lesions (13.5% vs 1.6%, respectively) but decreased TLR compared with that of RCA-AO lesions (36.1%) treated with bare-metal stents. TLR incidence was found to be higher in a larger retrospective study that compared 397 RCA-AO SES–treated patients with 3716 patients with non-ostial RCA lesions (28.2% vs 13.7%, respectively).^3^ Several studies have compared clinical outcomes after treating lesions with eDES with those after treating them with nDES. Watanabe et al^6^ found significantly reduced TLR rate using nDES compared with that using eDES for the treatment of RCA-AO lesions (12.7% vs 43.1%, respectively); most nDES in this study were everolimus-eluting stents (82.4%). In a larger study that retrospectively compared 192 RCA-AO lesions treated with nDES with 142 lesions treated with eDES, the rate of TLF at 3 years was significantly lower in the nDES group (14.2% vs 37.7%, respectively; *P* < .001), which was mainly driven by TLR (11% vs 38%, respectively; *P* < .001).^7^ Lam et al^4^ compared the outcomes of 67 patients with RCA-AO lesions with 254 patients with non-ostial RCA lesions treated with second-generation DES. During 2 years of follow-up, RCA-AO–treated patients recorded a higher incidence of TLR (7.5% vs 1.6%, respectively; *P* = .02). They concluded that the treatment of the ostial region of the RCA with second-generation DES is feasible; however, stent coverage of the right AO segment remains a predictor of TLR in RCA lesions.

PCI of narrowings involving the AO region is known to be technically more challenging because interventional location and guiding catheter engagement share the same space. AO lesions are often heavily calcified, fibrotic, and sclerotic^11^^,^^12^; this might implicate stent underexpansion or inhomogeneous expansion, which is a major risk factor of in-stent restenosis.^13^^,^^14^ The RCA ostium has significant elastic fibers owing to geometrical access from the aorta; therefore, elastic recoiling and stent fracture occur more frequently at this site.^11^^,^^15^

High radial strength in combination with high visibility and longitudinal stability of the device may be characteristics of an “ideal” stent for the treatment of AO lesions. In selected cases of acute AO stent recoiling, the so-called double stenting technique (ie, stent-in-stent implantation) has improved angiographic outcome.^16^

The high flexibility and thin-strut design of third-generation DES were associated with reduced longitudinal device stability,^17^ and it was uncertain whether these novel devices may indeed improve the outcome of AO lesion PCI. Our findings show that the use of this thin-strut (80.0 μm) cobalt-chromium SES is feasible and safe.

### Study limitations

Several potential limitations of this study should be noted. First, its observational nature may have affected the way these results are translated into clinical grounds. The baseline characteristics were balanced by IPSW; however, unmeasured variables with a potential impact on outcomes could not be included. Second, the low rates of predilation and postdilation in the study groups, low rates of intracoronary imaging of 6% to 7%, and absence of imaging core laboratory data for angiography and intravenous ultrasound implicate that it is unclear whether the increased TVR and stent thrombosis rates are related to a more suboptimal result or a higher risk of hyperplasia. Third, data regarding the PCI techniques used were absent.

The findings of this study apply to a specific DES platform and cannot, therefore, be extrapolated to other bioabsorbable or durable polymer DES platforms. The Ultimaster stent has an 80.0-μm strut thickness, which is higher than the 60.0-μm strut thickness of the Orsiro stents (Biotronic) in the stent of small diameters (<2.75 mm).

Continued divergence of outcomes might be seen after 1 year as stent failure rates accrue. However, even being nonrandomized, the e-Ultimaster registry was performed in 413 sites over 50 countries, so it represents up-to-date real-life data.

## Conclusions

Our study is the largest study to date to compare the outcomes of RCA-AO lesions with those of proximal RCA lesions. Our findings showed that RCA-AO treatment with currently available DES is feasible and was associated with numerically higher but statistically similar rates of the primary outcome of TLF, the composite of cardiac death, TVMI, and clinically driven TLR. We found significantly higher clinically driven TVR and stent thrombosis than those of non-ostial RCA lesions but similar mortality rate and MI rates. When considering RCA-AO treatment, there should be careful assessment of patient risk factors and the necessity of treatment.

## Declaration of competing interest

Bernard Chevalier reports grants from Terumo during the conduct of the study. Mamas A. Mamas reports an unrestricted educational grant from Terumo. Yaniv Levi, Ofer Kobo, Majdi Halabi, Imad Al Haddad, Jawed Polad, Peep Laanmets, Adam Witkowski, Jacques Monsegu, Andres Romo Iniguez, and Ariel Roguin reported no financial interests.

## Funding sources

The e-ULTIMASTER registry was funded by 10.13039/501100008645Terumo Europe, Middle East and Africa.

## Ethics statement and patient consent

The study was approved by the ethical committees of the participating sites, and all patients provided written informed consent.

## Supplementary material

Supplementary File (Word)
