# Supplementary material for: Treatment of Ostial Right Coronary Artery Narrowings: Outcomes From the Multicenter Prospective e-ULTIMASTER Registry
Source: J Soc Cardiovasc Angiogr Interv. 2023 Feb 25;2(3):100604. doi: 10.1016/j.jscai.2023.100604 (PMC11308816; doi:10.1016/j.jscai.2023.100604)
Supplement: Supplementary File (Word) [file mmc1.docx]

Supplement

**Supplemental Table 1: Definitions of primary outcome events**

| **Event** | **Definition** |
| --- | --- |
| Cardiac death | Cardiac death was defined as any death due to proximate cardiac cause (e.g. MI, low-output failure, fatal arrhythmia), un-witnessed death and death of unknown cause, and all procedure-related deaths, including those related to concomitant treatment. |
| Myocardial infarction | ).  Three types of myocardial infarction (MI) **depending of the** **time of occurrence:**   - **Pre-procedural MI**: occurring any time between the admission time at the hospital and the procedure. - **Peri-procedural MI**: occurring during the procedure or within 48h after procedure (baseline procedure or any repeated percutaneous coronary intervention) or 72h after CABG. Peri-procedural MI clearly related to revascularization of non-target vessel will not be counted as event for device oriented composite endpoint-TLF (see below) - **Spontaneous MI**: occurring any time beyond the baseline procedure and in between any repeated intervention (see below).   Two types of Myocardial infarction (MI) **depending on ECG assessment**:   - **Q-wave MI**: occurring with detection of development of new, pathological Q-waves in 2 or more contiguous leads. - **Non-Q-wave MI**: occurring in the absence of new pathological Q-waves, with elevation of values of cardiac enzymes CK and CK-MB and Troponin. In this protocol only Troponin T or Troponin I will be used for the assessment of myocardial infarctions, not high sensitivity troponin.   **PERIPROCEDURAL MI <48 HOURS after PCI**   1. New pathologic Q waves in ≥ 2 contiguous ECG leads ***AND***    - Any CKMB > 1*URL ***or***    - In the absence of CKMB: Troponin > 1*URL ***or***    - In the absence of CKMB and Troponin: CK > 1*URL ***or***    - In the absence of CKMB and Troponin and CK: CEC decision upon clinical scenario 2. Appropriate cardiac enzyme data (respecting top-down hierarchy, b1 to b3):   b1. CK ≥ 2*URL Confirmed by   - CKMB>1*URL ***or*** - In the absence of CKMB: Troponin > 1*URL ***or*** - In the absence of CKMB and Troponin: CEC decision upon clinical scenario   ***OR***  b2. In the absence of CK: CKMB >3*URL  ***OR***  b3. In the absence of CK and CKMB: Troponin >3*URL  Note URL = upper reference limit, defined as 99th percentile of normal reference range  **SPONTANEOUS MI > 48HOURS after PCI**   1. Recurrent thoracic chest pain or ischemic equivalent ***AND***   New pathologic Q waves in ≥2 contiguous ECG leads ***AND***:   - - Any CKMB >1*URL ***or***   - In the absence of CKMB: Troponin>1*URL ***or***   - In the absence of CKMB and Troponin: CK > 1*URL ***or***   - In the absence of CKMB and Troponin and CK: CEC decision upon clinical scenario  1. Appropriate cardiac enzyme data:   b1. CK ≥ 2*URL Confirmed by   - CKMB>1*URL ***or*** - In the absence of CKMB: Troponin>1*URL ***or*** - In the absence of CKMB and Troponin: CEC decision upon clinical scenario   ***OR***  b2. In the absence of CK: CKMB >3*URL  ***OR***  b3. In the absence of CK and CKMB: Troponin >3*URL  ***OR***  b4. In the absence of CK, CKMB and Troponin, clinical decision based upon clinical scenario. |
| Target vessel myocardial infarction | Myocardial infarction that could not be clearly attributable to a vessel other than target vessel(s) |
| Clinically-driven target lesion revascularization | Target lesion revascularization (TLR) is any clinically driven repeat percutaneous intervention of the target lesion or bypass surgery of the target vessel performed for restenosis or other complication of the target lesion. All TLRs were classified prospectively as clinically indicated or not clinically indicated by the Investigator prior to repeat angiography. The target lesion was defined as the treated segment from 5 mm proximal to the stent and to 5 mm distal to the stent.  A revascularization was considered clinically driven if angiography at follow-up showed a percent diameter stenosis≥ 50% (core lab QCA assessment) and if one of the following occurred: 1. a positive history of recurrent angina pectoris, presumably related to the target vessel; 2. objective signs of ischemia at rest (ECG changes) or during exercise test (or equivalent),presumably related to the target vessel; 3. abnormal results of any invasive functional diagnostic test (e.g. Doppler flow velocity reserve, fractional flow reserve); 4. diameter stenosis≥ 70% (by corelab QCA assessment) even in the absence of the above-mentioned ischemic signs or symptoms. |

MI: myocardial infarction; PCI: percutaneous coronary intervention; TLR: target lesion revascularization

**Supplemental Table 2: Clinical Event Committee members**

| **Name** | **Affiliated hospital** |
| --- | --- |
| Taku Asano | St Luke’s International Hospital, Tokyo, Japan |
| Claude Hanet | Catholic University Hospital Mont-Godinne, Belgium |
| Hara Hironori | Academic Medical Center (AMC), Amsterdam, the Netherlands |
| Yuki Katagiri | Academic Medical Center (AMC), Amsterdam, the Netherlands |
| Hideyuki Kawashima | Academic Medical Center (AMC), Amsterdam, the Netherlands |
| Norihiro Kogame | Academic Medical Center (AMC), Amsterdam, the Netherlands |
| Hidenori Komiyama | Nippon Medical school, Tokyo, Japan |
| Yosuke Miyazaki | Erasmus Medical Center Rotterdam, the Netherlands |
| Masafumi Ono | Academic Medical Center (AMC), Amsterdam, the Netherlands |
| Bastiaan Schölzel | Amphia Ziekenhuis Breda, the Netherlands |
| Kuniaki Takahashi | Academic Medical Center (AMC), Amsterdam, the Netherlands |
| George Vlachojannis | Maasstad Ziekenhuis Rotterdam, the Netherlands |

**Supplemental Table 3: List of participating sites and investigators**

ARGENTINA: Fundación Favaloro: Oscar Mendiz; Hospital Universitario Austral: Juan Manuel Telayna; Clinica Centro Médico Privado Junin: José Magni; Instituto Cardiovascular de Buenos Aires: Fernando Cura; Sanatorio San Miguel: Juan Lloberas; ARMENIA: Astghik Medical Center (Natali Farm): Mikayel Adamyan; Medical Center Gyumri CJSC: Davit Minasyan; Qancor Cardiovascular MC LLC: Shahen Khachatryan; Republican Medical Center Armenia CJSC: Boghos Sarkissian; Yerevan State Medical University Hospital: Hamayak Sisakian; AUSTRIA: AKH Linz: Clemens Steinwender; Medical University Vienna (AKH): Irene Lang; Medizinische Universität Graz: Gabor Toth-Mayor; BANGLADESH: National Heart Foundation Hospital and Research Institute: Fazila Tun-Nesa Malik; BELARUS: City Clinical Emergency Hospital: Alexander Beimanov; RSPC: Oleg Polonetsky; BELGIUM: AZ Sint Lucas: Jan Nimmegeers; CHR de La Citadelle: Suzanne Pourbaix; Hôpital Ambroise Paré de Mons: Stéphane Carlier; CHU Charleroi: Adel Aminian; CHU UCL Mont Godinne Namur: Antoine Guédès; Epicura Hornu: Philippe Decroly; Imelda Ziekenhuis: Willem De Wilde; Jan Yperman Ziekenhuis: Dries De Cock; OLVZ Aalst: Bernard De Bruyne; UCL Saint Luc: Joelle Kefer; BRAZIL: Eurolatino Natal Pesquisas Medicas (Eurolatino Natal Medical Research): Maria Sanali Paiva; Hospital E Maternidade Dr. Christóvão Da Gama: Bruno Palmieri Bernardi; Hospital Felicio Rocho: Jamil Abdalla Saad; Hospital Moinhos de Vento: Marco Vugman Waistein; Hospital Monte Sinai: Gustavo De Moraes Ramalho; Hospital Santa Cruz: Roberto Otsubo; Hospital São Vicente de Paulo: Rogério Tumelero, Alexandre Tognon; Paraná Medical Research Center: Marcos Franchetti; Unicor: João Eduardo Tinoco De Paula; Unimed Joinville: Bruno Cupertino Migueletto; BULGARIA: Mbal Haskovo: Sevdalin Topalov; Mbal Montana City Clinic Sveti Georgi: Krasimir Pandev; Mbal Sveta Karidad, Plovdiv: Dimitar Karageorgiev; Mbal Sveta Petka Vidin: Diana Trendafilova-Lazaroba; Specialized Cardiology Hospital For Active Treatment: Angel Mitov; Trakiya Hospital, Stara Zagora: Borislov Borisov; Umhat Alexandrovska: Dobrin Vassilev; Umhat St.Ekaterina: Julia Jorgova-Makedonska; CHILE: Clinica Bicentenario: Carlos Romero; Clinica Santa Maria: Pablo Pedreros; Hospital Clínico San Borja Arriaran: Gabriel Maluenda; Hospital Guillermo Grant Benavente: Luis Perez; Hospital Regional de Antofagasta: Bernhard Westerberg; Hospital Regional Puerto Montt: Victor David Assef; Hospital San Juan de Dios: Angel Puentes; COLOMBIA: Centro Cardiovascular de Caldas: Hugo Castaño; Clinica Shaio: Pablo Castro; Fundación Cardiovascular de Colombia (Bucaramanga): Tamara Gorgadze; Instituto del Corazon Bucaramanga: Boris Eduardo Vesga, Hector Hernandez; CZECH REPUBLIC: St Anne’s University Hospital Brno: Ladislav Groch; Kardiologie na Bulovce: Miroslav Erbrt; Karlovarská Krajská Nemocnice: Alexandr Schee; FNKV Hospital: Viktor KočKa; Krajska Nemocnice T. Bati: Zdenek Coufal; EGYPT: Al Hayat Hospital: Hany Ragy; Al Nakheel Hospital: Yasser Sadek; Dr Ahmed Abdel Aziz Multicenter: Mohamed Abdel Aziz; Dr Hussien Heshmat – As Salam International Hospital: Hussien Heshmat; El Marwa Hospital: Mounir Asman; Italian Hospital: Ihab Daoud; L-Fouad Cardiac Center: Ahmed Emara; Dr Hisham Ammar Multicenter: Hisham Ammar; Police Hospital: Mohamed Helal; Dr. Tarek Rasid: Tarek Rashid; Um El Korra M Setiha Hospital: Mohamed Setiha; Nile Badrawy Hospital: Sameh Ahmed Salama; Wadi El Neel: Hazem Khamis; ESTONIA: North-Estonia Medical Center: Peep Laanmets; FRANCE: Centre D'exploration-Chirurgie Cardio-Vasculaire: Jean-Louis Leymarie; CH Bretagne Atlantique: Emmanuelle Filippi; CH de Marne La Vallée: Simon Elhadad; CH de Montreuil: Chaib Aures; CH Haguenau: Fabien De Poli; Groupe Hospitalier de la Rochelle Ré Aunis: Charlotte Trouillet; CH La Timone Marseille: Jean-Louis Bonnet; CH Louis Pasteur-Le Coudray: Grégoire Rangé; CH de Pau: Nicolas Delarche; CH René Dubos Pontoise: Francois Funck; CH St Joseph St Luc Lyon: Olivier Dubreuil; CH Sud Francilien: Pascal Goube; CH Valence: Stanislas Champin; CH Yves Le Foll - Saint Brieuc: Denis Amer Zabalawi; CHD Vendée La Roche Sur Yon: Emmanuel Boiffard; CH Général de Saint Quentin: Pierre Henon, Florent Chevalier; CHIC Quimper: Thierry Joseph; CHR Orleans Cardiologie: Olivier Bizeau; CHU Angers: Alain Furber; CHU Caen: Farzin Beygui; CHU Clermont-Ferrand: Pascal Motreff; CHU de Poitiers: Sebastien Levesque; Clinique Ambroise Paré: Julien Rosencher; Clinique Diaconat Fonderie Mulhouse: Pradip Kumar Sewoke; Clinique du Millénaire Montpellier: Christophe Piot; Clinique Du Pont de Chaume Montauban: Laurent Delorme; Clinique Louis Pasteur Essey les Nancy: Max Amor; Clinique Rhône Durance: Gilles Bayet; Clinique Saint-Laurent: Yves Biron; Clinique St Hilaire Rouen: Matthieu Godin; Clinique St Joseph: Julien Jeanneteau; GCS Cardiologique de Bayonne: Jean Luc Banos; Groupe Hopitalier Paris Saint Joseph: Romain Cador; Groupement Mutualiste de Grenoble: Jacques Monsegu; Hopital Privé Claude Galien Quincy: Stéphane Champagne; Hopital Albert Schweitzer GHCA Colmar: Plastaras Philoktimon; Hôpital Europøen de Paris la Roseraie: Hakim Benamer; Hopital Privé Dijon Bourgogne: Philippe Brunel; Hopital Privé Jacques Cartier Massy: Thomas Hovasse; Hopital Privé La Louviere-Lille: Fabrice Leroy; Hopital Privé Saint Martin: Guillaume Lecoq; Hôpital Privé St Martin de Pessac: Levy Raphy; Hôpital Privé St Martin de Pessac: Bernard Karsenty; Institut Arnault Tzanck St Laurent du Var: Alexandre Avran; Le Confluent Nouvelles Cliniques Nantaises: Ashok Tirouvanziam; Nouvel Hopital Civil de Strasbourg: Olivier Morel; Pôle Santé République Clermont Ferrand: Pascal Barraud; Polyclinique Les Fleurs: Philippe Commeau; GEORGIA: Joann Medical Center (JAMC): Lasha Chantladze; HUNGARY Pándy Kálmán Hospital: Jambrik Zoltan; Markusovszky University Teaching Hospital: Lajos Nagy; Moritz Kaposi General Hospital: Andras Vorobcsuk; PECS University: Ivan Horvath; Semmelweis University: Bela Merkely; Szabolcs - Szatmar - Bereg County Hospital and University Teaching Hospital: Kôszegi Zsolt; ICELAND Landspitali National University Hospital of Iceland: Ingibjörg Jóna Guðmundsdóttir; INDIA Dayanand Medical College: Gurpreet Singh Wander; Fortis Hospital: R. Keshava; G. Kuppuswamy Naidu Memorial Hospital: Rajpal Abhaichand; H.J. Doshi Ghatkopar Hindusabha Hospital: Anil Potdar; Heart & General Hospital: Prakash Chandwani; Kamalnayan Bajaj Hospital, Aurangabad: Ajit Bhagwat; Krishna Institute of Medical Sciences: Rajendra Kumar Premchand; Madras Medical Mission: Ajit Mullasari; Maharaja Agrasen Hospital: B B Chanana; Max Super Specialty Hospital: Viveka Kumar; Medanta Hospital: Praveen Chandra; BM Birla Heart Research Cente: Ashwani Mehta; Sree Chitra Tirunal Institute of Medical Sciences & Technology: Bijulal Sasidharan; Wockhardt Hospital: Prashant Jagtap; INDONESIA Awal Bros Hospital: Bambang Budiono; Binawaluya Cardiac Center: Muhammad Munawar; RSUPN Dr. Cipto Mangunkusumo Hospital: Muhammad Yamin; Dr. Soetomo General Hospital: Yudi Her Oktaviono; Dr. Wahidin Sudirohusodo General Hospital- Awal Bros Hospital: Abdul Hakim Alkatiri; Medistra Hospital: Teguh Santoso; National Cardiovascular Center Harapan Kita Hospital: Doni Firman; Saiful Anwar General Hospital: Sasmojo Widito; IRELAND Cork University Hospital: Eugene McFadden; University Hospital Galway: Jim Crowley; University Hospital Limerick: Thomas Kiernan; ISRAEL Assaf Harofeh Medical Center: Minha Saar; Galilee Medical Center: Marc Brezins; Rambam Medical Center: Ariel Roguin; Ziv Medical Center: Majdi Halabi; JAPAN Gunma Prefectural Cardiovascular Center: Ren Kawaguchi; Higashi Takarazuka Satoh Hospital: Satoru Otsuji; Iwaki Kyoritsu General Hospital: Yoshito Yamamoto; Kakogawa Central City Hospital: Makoto Kadotani; Kansai Rosai Hospital: Takayuki Ishihara; Kokura Memorial Hospital: Kenji Ando; Komaki City Hospital: Katsuhiro Kawaguchi; Kouseikai Takai Hospital: Yasunori Nishida; Mie Heart Center: Hideo Nishikawa; Mimihara General Hospital: Shozo Ishihara; Okamura Memorial Hospital: Yasuhiro Tarutani; Osaka General Medical Center: Takashi Morita; Osaka Rosai Hospital: Masami Nishino; Saiseikai Senri Hospital: Keiji Hirooka; Saiseikai Yamaguchi General Hospital: Shiro Ono; Saiseikai Yokohama City Eastern Hospital: Yoshiaki Ito; Saitama Cardiovascular And Respiratory Center: Makoto Muto; Sakurabashi Watanabe Hospital: Kenshi Fujii; Sapporo Higashi Tokushukai Hospital: Seiji Yamazaki; Seirei Hamamatsu General Hospital: Hisayuki Okada; Seirei Yokohama Hospital: Kazuhiro Ashida; Shonan Kamakura General Hospital: Shigeru Saito; Showa University Fujigaoka Hospital: Hiroshi Suzuki; Tokai University Hachiouji Hospital: Takashi Matsukage; JORDAN Jordan Hospital: Imad Alhaddad; KAZAKHSTAN Aktobe Regional Hospital: Aidos Taumov; Cardiology Center Petropavl: Maxat Kudratullayev; City Hospital #2: Marat Alikhanov; Clinical Center of Cardiac Surgery and Transplantation: Vadim Seisembekov; Jsc Nat. Scient. Cardiosurgery Ctr.: Marat Aripov; Medical University Clinic West Kazakhstan: Dauren Teleuov; National Surgery Center Almaty: Bauyrzhan Ormanov; Pavlodar Regional Cardiologic Center: Ruslan Baisebenov; Regional Cardiosurgery Center: Azamat Kenzhinovich Zhashkeyev; Rudnyi City Hospital: Azamat Yerzhanov; The Almaty City Heart Center: Orazbek Sakhov; Semey State Medical University, Interventional Cardiology Dpt: Ersin Sabitov; KUWAIT Sabah Al Ahmad Cardiac Center: Vladimir Kotevski; LEBANON Hôpital Abou Jaoudé: Daou Abdo; Labib Medical Center: Ahmad Serhal; LITHUANIA Hospital Of Lithuanian University Of Health Sciences Kauno klinikos: Ramunas Unikas; Klaipeda Seamen's Hospital: Aurimas Knokneris; MACEDONIA City General Hospital: Vladimir Ristovski; University Clinic Of Cardiology: Sasko Kedev; MALAYSIA Desa Park City: Chong Yoon Sin; Hospital Serdang: Abdul Kahar Ghapar; Hospital Sultanah Bahiyah: Abd Syukur Bin Abdullah; Hospital Tengku Ampuan Afzan: Siti Khairani bt Zainal Abidin; HSC Medical Center: Tee Chee Hian; UiTM Sg. Buloh Campus: Nicholas Chua Yul Chye; MEXICO Clinica Hospital San Jose de Navojoa: Santiago Sandoval Navarrete; Hospital Fray Juan de San Miguel de Uruapan: Juan Jorge Beltran Ochoa; Hospital Star Medica Merida: Sergio Alonso Villareal Umaña; Casa del Corazon de la Peninsula de Yucatan SCP: Carlos Ramon Rodas Caceres; MOROCCO Cherradi_Clinique Agdal: Rhizlan Cherradi; Clinique Achifaa de Casablanca: Anass Assaidi; Clinique Grant Atlas: Dounia Benzaroual; Clinique Internationale de Marrakech: Fahd Chaara; NETHERLANDS Albert Schweitzer Ziekenhuis: Martijn Scholte; Amphia Ziekenhuis: Alexander J.J. Ijsselmuiden; Catharina Ziekenhuis: W.A.L. Pim Tonino; Jeroen Bosch Ziekenhuis: Jawed Polad; Jacob van Eck; Maasstad Ziekenhuis: Pieter Cornelis Smits; Meander MC: Fabrizio Spano; Medisch Centrum Haaglanden: Lucas H. Savalle; Medisch Spectrum Twente, Enschede: Clemens Von Birgelen; Rijnstate Ziekenhuis: Peter W. Danse; Scheper Hospital: Gillian Jessurun; Zorgsaam Ziekenhuis Zeeuws-Vlaanderen: Pieter Bisschops; OMAN Muscat Private Hospital: Amr Hassan; POLAND Insytut Kardiologii im. Prymasa Tys ąclecia Stefana Kardynała Wyszyńskiego: Adam Witkowski; Miedziowe Centrum Zdrowia: Adrian Wlodarczak; Szpital Kliniczny Przemienienia Panskiego Um Im. K. Marcinkowskiego W Poznaniu: Maciej Lesiak; PORTUGAL CHLN Norte Hospital Santa María: Pedro Canas Da Silva; ROMANIA Centrele de Excelenta Ares: Alexandru Voican; Clinicile Icco S.R.L.: Mihai Ursu; Cordismed Timisoara: Milovan Slovenski; Spitalul Judetean de Urgenta Sibiu: Ioan Bitea Cornel; SAUDI ARABIA Dallah Hospital, Riyadh: Samih Lawand; King Fahad Cardiac Center: Tarek Kashour; Prince Abdullah Bin Abdul Aziz Musad Cardiac Center: Muhammad Aurangzaib Mughal; SERBIA Cardiovascular Institute Dedinje: Dragan Sagic; Clinical Center Kragujevac: Nikola Jagic; Cardiology Clinic, Clinical Centre of Serbia: Vladan Vukcevic; Kbc Zvezdara: Alexandar Davidovic; CHC Bezanijska Kosa: Sasa Hinic; SLOVAKIA Stredodlovensky Ustav Srdcovych A Cievnych Chorob: Martin Hudec; SOUTH AFRICA Ethekwini Hospital & Heart Centre: Shiraz Gafoor; Ismail Soosiwala; Milpark Hospital: Graham Cassel; Netcare Greenacres Hospital: Martin Tawanda Butau; Netcare Union Hospital: Jean-Paul Theron; Netcare Unitas Hospital: Jean Vorster; Netcare Unitas Hospital: Pieter Blomerus; Netcare Unitas Hospital: Iftikar Osman Ebrahim; Netcare Unitas Hospital: Jacobus Badenhorst; SPAIN Bellvitge University Hospital: Joan Antonio Gomez; Complexo Hospitalario Universitario A Coruña (CHUAC): Nicolás Vázquez Gonzalez; Hospital 12 Octubre: Fernando Sarnago; Hospital Cabueñes: Iñigo Lozano; Hospital Clínico Lozano Blesa de Zaragoza: José Ramón Ruiz Arroyo; Hospital Clínico Universitario de Santiago de Compostela: Ramiro Trillo Nouche; Clinico Universitario Valencia: Juan Sanchís; Hospital de Cruces-Barakaldo: Juan Alcibar; Hospital Universitario Donostia: Mariano Larman; Hospital de Galdakao: José Ramón Rumoroso; Hospital de La Cruz Roja de Córdoba: José Suárez de Lezo; Hospital de León: Maria López Benito; Hospital de Mérida: Pablo Cerrato Garcia; Hospital de Navarra: Baltasar Lainez; Hospital del Mar: Beatriz Vaquerizo; Hospital Fundacion Alcorcon: Javier Botas; Hospital G. Trias I Pujol: Eduard Fernández Nofrerias; Hospital General Castellón: Pascual Baello Monge; Hospital General Ciudad Real: Fernando Lozano Ruiz-Poveda; Hospital General de Albacete: Jesus Maria Jimenez Mazuecos; Hospital General Universitario de Burgos: Javier Robles; Hospital Infanta Cristina: José Ramon Lopez Minguez; Hospital Juan Ramón Jiménez: Pepi Garcia; Clinica La Luz: Jorge Palazuelos; Hospital Manises: Gema Miñana; Hospital Marqués de Valdecilla: Jose Javier Zueco; Hospital Meixoeiro-Medtec: Andrés Iñiguez Romo; Hospital Moncloa: Eulogio Garcia Fernandez; Hospital Puerta de Hierro: Javier Goicolea; Hospital Reina Sofia de Córdoba: Manuel Pan; Clínica San Fransisco de Asis: Arturo García Touchard; Hospital San Pedro: Javier Fernández; Hospital San Pedro de Alcantara-Caceres: Javier Fernandez Portales; Hospital San Rafael: Gonzalo Peña; Hospital Sant Pau: Antonio Peñaranda Serra; Hospital Santa Lucía de Cartagena Hospital Nostra Señora Rossell: José Domingo Cascón; Hospital Txagorritxu: Alfonso Torres; Hospital Universitario de Gran Canaria Dr Negrin: Pedro Martin Lorenzo; Hospital Universitario de Guadalajara: Javier Balaguer Requena; Hospital Universitario Lucus Augusti (HULA): Raymundo Ocaranza Sanchez; Hospital Universitario Miguel Servet (H.U.M.S.): Jose Antonio Diarte de Miguel; Hospital Vall d'Hebron: Bruno García Del Blanco; Hospital Virgen Arrixaca: Eduardo Pinar; Hospital Virgen de La Salud: P. José Moreu Burgos; Instituto Cardiologico Hospital Campo Grande: Juan Manuel Duran; San Juan de Alicante: Ramón López Palop; Universitario Central de Asturias: César Moris-De La Tassa; SWEDEN Gävle Sjukhus: Robert Kastberg; Mälarsjukshuet: Finn Hjortevang; Skaraborgs Sjukhus v Skövde: Jason Stewart; Sundvalls Sjukhus: Espen Haugen; Universitets Sjukhuset I Örebro: Ole Fröbert; Västmanlads Sjukhus Västerås: Amra Kåregren; SWITZERLAND Cardiocentro Lugano, Ticino: Giovanni Pedrazzini; Herz Gefäss Zentrum Zürich: Peter Wenaweser; Hôpital de La Tour: Edoardo De Benedetti; Hôpitaux Universitaires de Genève: Marco Roffi; Kantonsspital Baselland: Gregor Leibundgut; Kantonsspital Frauenfeld Spital Thurgau AG: Michael Neuhaus; Kantonsspital Luzern: Florim Cuculi; THAILAND Central Chest Institute Of Thailand: Wirash Kehasukcharoen; HRH Princess Maha Chakri Sirindhorn Medical Center (Nakornayok): Arthit Wongsoasup; Paolo Memorial Hospital Phaholyothin: Niphonth Srisuwanunt; TUNISIA Dr. Mohamed Drissa Clinique Hannibal Lac 2: Mohamed Akram Drissa; Dr. Ben Chedli Tarek - Soukra Medical: Ben Chedli Tarek; Dr. Bouziri - Clinique Générale Et Cardiovasculaire de Tunis: Sami Bouziri; Dr. Elyes Kharrat - Bassatine Clinic: Elyes Kharrat; Polyclinique El bassatine_Dr. Mohamed Najeh Abid: Mohamed Najeh Abid; Clinique Générale et Cardiovasculaire de Tunis _Dr. Saloua Trabelsi: Saloua Trabelsi; Polyclinique El Bassatine: Rridha Ennouri; UKRAINE Heart Institute: Andriy Khohlov; NAMS Amosov | Emergency Endovascular Surgery Department: Sergii Salo; NAMS Amosov | X-Ray Diagnostics And Invasive Cardiology Department: Yevhenii Aksonov; S.P.M.C. of Pediatric Cardiology and Cardiac Surgery: Georgiy Mankovskiy; UNITED ARAB EMIRATES Al Noor Hospital - Airport: Mohammad Andron; Al Qassimi Hospital: Arif Al Nooryani; Al Zahra Private Hospital, Dubai: Syed Nazir; Belhoul Speciality Hospital, Dubai: Muhammad Adnan Raufi; Dr. Sulaiman Al Habib: Albert Alahmar; Dubai Hospital: Hesham Ahmed Osman; Iranian Hospital, Dubai: Seyed Bagher Tabatabaei; Lifecare Hospital: Khaled Galal; Prime Hospital, Dubai: Murali Krishna; Rashid Hospital: Fahad Omar Baslaib; UNITED KINGDOM Essex Cardiothoracic Centre, Basildon: Rohan Jagathesan; Bedford Hospital: Ramesh de Silva; Blackpool Victoria Hospital: Jonas Eichhofer; Bradford Teaching Hospitals: John Kurian; Croydon University Hospital: Sanjay Kumar; Dorset County Hospital: Javed Iqbal; Eastbourne District General Hospital: David Walker; Freeman Hospital: Rajiv Das; GBS Re Bucks Healthcare NHS Trust (Buckinghamshire, Wycombe): Piers Clifford; James Cook University Hospital: David Austin; Kettering General Hospital: Javed Ehtisham; Kings Mill Hospital: Ifti Fazal; Lincoln County Hospital: Kelvin Lee; Lister Hospital, Stevenage: Paul Kotwinski; The Royal Wolverhampton Hospitals: Shahzad Munir; Norfolk And Norwich University Hospital: Alisdair Ryding; Northwick Park Hospital: Ahmed Elghamaz; Plymouth Hospital: Girish Viswanathan; Queen Elizabeth Hospital, Birmingham: Sagar Doshi; Queens Medical Center Nottingham: Sachin Jadhav; Royal Berkshire Hospital: Nicos Spyrou; Royal Blackburn Hospital: John Mcdonald; Royal Bournemouth And Christchurch Hospitals NHS Foundation Trust: Suneel Talwar; Royal Brompton And Harefield: Robert Smith; Royal Cornwall Hospitals: Sen Devadathan; Derby Teaching Hospitals: Kamal Chitkara; The Royal Free Hospital: Sundeep Kalra; Royal Gwent Hospital, Newport: James Cullen; Royal Stoke University Hospital: Mamas Mamas; Royal Sussex Hospital, Brighton: David Hildick Smith; Royal United Hospital, Bath: Kevin Carson; Salisbury District Hospital: Tim Wells; Sandwell And West Birmingham Hospitals: Chetan Varma; Sheffield Teaching Hospital: James Richardson; Tunbridge Wells Hospital: Clive Lawson; UH Coventry and Warwickshire: Rajathurai Thirumaran; University Hospital South Manchester: Hussain Contractor; University Hospital Of Wales: Rito Mitra; University Hospitals Of Leicester: Ian Hudson; West Middlesex Hospital: Sukhinder Nijjer; Western Sussex Hospitals - Worthing Hospital: Nicholas Pegge; Worcestershire Acute Hospitals NHS Trust: Helen Routledge; Wrightington Hospital: V J Karthikeyan; UZBEKISTAN Republic Specialized Center of Surgery: Mirjamol Mirumarovich Zufarov; VIETNAM Thong Nhat Hospital: Nguyen Van Tan
